# Editorial Note: Early Growth Response Gene-2 (Egr-2) Regulates the Development of B and T Cells

**DOI:** 10.1371/journal.pone.0362246

**Published:** 2026-11-06

**Authors:** The PLOS One Editors

The *PLOS One* Editors issue this Editorial Note to inform readers that concerns have been raised about results presented in Fig 5A (Lymph node panels) of this article [1].
